# Levels of cross-resistance to pyrethroids conferred by the *Vssc knockdown resistance* allele 410L+1016I+1534C in *Aedes aegypti*

**DOI:** 10.1371/journal.pntd.0009549

**Published:** 2021-07-12

**Authors:** Juan J. Silva, Cedric N. Kouam, Jeffrey G. Scott

**Affiliations:** Department of Entomology, Comstock Hall, Cornell University, Ithaca, New York, United States of America; Connecticut Agricultural Experiment Station, UNITED STATES

## Abstract

*Aedes aegypti* is a primary vector of viral pathogens and is responsible for millions of human infections annually that represent critical public health and economic costs. Pyrethroids are one of the most commonly used classes of insecticides to control adult *A*. *aegypti*. The insecticidal activity of pyrethroids depends on their ability to bind and disrupt the voltage-sensitive sodium channel (VSSC). In mosquitoes, a common mechanism of resistance to pyrethroids is due to mutations in *Vssc (*hereafter referred as *knockdown resistance*, *kdr)*. In this study, we found that a *kdr* (410L+V1016I+1534C) allele was the main mechanism of resistance in a pyrethroid-resistant strain of *A*. *aegypti* collected in Colombia. To characterize the level of resistance these mutations confer, we isolated a pyrethroid resistant strain (LMRKDR:RK, LKR) that was congenic to the susceptible Rockefeller (ROCK) strain. The full-length cDNA of *Vssc* was cloned from LKR and no additional resistance mutations were present. The levels of resistance to different pyrethroids varied from 3.9- to 56-fold. We compared the levels of resistance to pyrethroids, DCJW and DDT between LKR and what was previously reported in two other congenic strains that share the same pyrethroid-susceptible background (the ROCK strain), but carry different *kdr* alleles (F1534C or S989P + V1016G). The resistance conferred by *kdr* alleles can vary depending on the stereochemistry of the pyrethroid. The 410L+1016I+1534C *kdr* allele does not confer higher levels of resistance to six of ten pyrethroids, relative to the 1534C allele. The importance of these results to understand the evolution of insecticide resistance and mosquito control are discussed.

## Author summary

*Aedes aegypti* is a primary vector of viral pathogens and is responsible for millions of human infections and pyrethroids are one of the most commonly used classes of insecticides to control adult *A*. *aegypti*. The insecticidal activity of pyrethroids depends on their ability to bind and disrupt the voltage-sensitive sodium channel (VSSC). A common mechanism of resistance to pyrethroids is due to mutations in *Vssc (*hereafter referred as *knockdown resistance* mutations, *kdr)*. In this study, we found that a *kdr* (410L+1016I+154C) allele was the main mechanism of resistance in a pyrethroid-resistant strain of *A*. *aegypti* collected in Colombia. We determined the levels of resistance to pyrethroids, DCJW and DDT conferred by this allele. The 410L+1016I+1534C *kdr* allele does not confer higher levels of resistance to six out of 10 pyrethroids relative to the 1534C allele. Resistance conferred by *kdr* alleles can vary between pyrethroids and can vary between the stereoisomers. Knowing the levels of resistance conferred by different alleles to different insecticides provides valuable information for pest control and for understanding the evolution of resistance.

## Introduction

*Aedes aegypti* is the primary vector of viral pathogens including chikungunya, dengue, yellow fever, and Zika and is responsible for millions of human infections annually that results in a critical threat for public health. Chikungunya has caused more than 440,000 cases of disease in more than 20 countries throughout the Americas (excluding North America) and the Caribbean by 2014 [1]. According to the Global Burden of Disease Study, dengue is estimated to cause approximately 58.4 million annual symptomatic cases resulting in 10,000 deaths per year [2]. Yellow fever is endemic to 47 countries and is responsible for 60,000 deaths annually [3]. Zika has been a public health problem due to its fast dispersion primarily in the Americas and the Caribbean [4,5]. It is estimated that Zika virus infected 440,000 to 1,300,000 people in South America by 2016 [6].

Pyrethroid insecticides are widely used to control adult *A*. *aegypti*, and the insecticidal activity of pyrethroids depends on their ability to disrupt the voltage-sensitive sodium channel (VSSC). Previous studies have characterized the importance of pyrethroid stereochemistry to insecticidal activity. These studies found differential toxicity between enantiomers (*R* or *S* conformations of the chiral carbons of the cyclopropane ring and α-cyano group) and diastereomers (*cis/trans* isomers of the cyclopropane ring). The 1*R* conformation of the cyclopropane ring is generally more toxic than the 1*S* conformation [7–10]. The *S* conformation of the *α*-cyano group (*αS*) usually has higher insecticidal activity than the *αR* conformation [8,11]. The *cis* isomers of phenoxybenzyl pyrethroids are usually more toxic to insects than *trans* isomers [12]. It was found that 1*R-cis αS* phenoxybenzyl pyrethroids (e.g. deltamethrin) often have higher insecticidal activity relative to the other conformations (e.g. 1*S-cis αS*, 1*R-trans αS*, etc.) [7,13].

In mosquitoes, a common mechanism of resistance to pyrethroids is target-site mutations in *Vssc (*hereafter referred as *knockdown resistance* mutations or *kdr)*. The *Vssc* mutations that have been found in *A*. *aegypti* are V235F, V410L, G923V, L982W, S989P, I1011V/M, V1016G/I, T1520I, F1534C and D1763Y (amino acid numbering based on *Musca domestica* VSSC, GenBank: CAA65448.1) [14]. In addition, the following alleles have been found in *A*. *aegypti*: 410L+1534C [15], 410L+1016I+1534C [16], 989P+1016G [17], 989P+1016G+1534C [18], 1520I+1534C [19], 1011M+923V [20], 1016I+1534C [21], 253F+1534C [22] and 1016G+1763Y) [23]. The two most common *kdr* alleles in Asia are 1534C and 989P+1016G, while the most common alleles in the Americas are 410L+1016I+1534C, 410L+1534C and 1534C [24]. Previous studies using congenic strains of *A*. *aegypti* found that the 989P+1016G allele conferred 21- to 107-fold resistance to multiple pyrethroids [25]. Another study found that 1534C allele conferred only 7- to 16-fold resistance to pyrethroids [24]. Among *Vssc* mutations, only a few (individually or in combination) have been shown to alter pyrethroid sensitivity based on heterologous expression in *Xenopus* oocytes [15,26–28]. Relative to the V410L, V1016I and F1534C mutations, permethrin insensitivity ranked 410L+1534C > 410L > 1534C [15] and deltamethrin insensitivity ranked 410L+1534C = 410L [15] with no insensitivity conferred by 1534C [15,26–28]. In contrast, the F1534C allele gives ~10-fold resistance to both permethrin and deltamethrin in adult *A*. *aegypti* [24].

In this study, we found that the major mechanism of resistance to pyrethroids in the LM-R strain of *A*. *aegypti* collected in Colombia was *kdr* (410L+1016I+1534C). We isolated a strain that was congenic to the susceptible strain, but carried these *kdr* mutations (LMRKDR:RK, LKR). We examined the resistance levels in LKR to 20 insecticides that target the VSSC. Our results were compared to what was previously reported for two other *kdr* alleles (989P+1016G and 1534C), as well as what was reported using heterologous expression in oocytes. The importance of these results to understanding the evolution of insecticide resistance and improving mosquito control is discussed.

## Materials and methods

### *A*. *aegypti* strains and rearing conditions

Four strains of *A*. *aegypti* were used in this study: Rockefeller (ROCK) is an insecticide susceptible strain, KDR:ROCK is a pyrethroid-resistant strain which is congenic to ROCK, but contains the *kdr* allele 989P+1016G [25], 1534C:ROCK is a pyrethroid-resistant strain which is congenic to ROCK, but contains the *kdr* allele F1534C [24], and La Mesa (LM). The LM strain was from eggs that were collected in ovitraps according to the protocol of the World Health Organization [29] in April 2016 in the town of La Mesa, Colombia (04° 38’ 09” N, 74° 27’ 59” W) and sent to Cornell University. The La Mesa (LM) strain was then selected with cyhalothrin for three consecutive generations (as described in the next section).

Mosquitoes were reared in cages (35 X 25 X 25 cm) with one Erlenmeyer flask containing 10% sugar water. Adult females were blood-fed using a membrane-covered water-jacketed glass feeder with cow blood (Owasco Meat Co Inc, Owasco NY) containing 0.3% sodium citrate to prevent clotting. Larvae were reared in containers (27.5 X 21.5 X 27.5 cm) with approximately 2 L of distilled water and fed with Cichlid Goldfish food pellets (Hikari Sales USA Inc., Hayward CA). Mosquitoes were reared at 27 ± 1°C and 50–60% relative humidity.

### Selection of the LM-R strain

We selected (with cyhalothrin) 3-7-day old adult mosquitoes from the LM strain over three consecutive generations to increase the frequency of resistance alleles in the strain. For each generation, an average of 2,790 unmated females and 1,164 males were treated with cyhalothrin. Mosquitoes were knocked down with CO_2_ and placed in a paper cup on a bucket filled with ice to keep them anesthetized for approximately 10 minutes. Insecticide doses were applied topically to individual mosquitoes with a PB600-1 repeating dispenser and a 10 μL Hamilton syringe (Hamilton Company, Reno NV). The selection doses were designed to kill ~80% of the individuals. Following cyhalothrin applications, 40 mosquitoes were placed in a paper cup covered with nylon tulle and a cotton ball saturated with distilled water and held at 25°C for 24 hours. Both male and unmated female survivors were released in cages and allowed to mate *en masse*. The resulting strain is referred as LM-R. This strain was tested for resistance due to enzyme-mediated detoxification using insecticide synergists (section Adult female bioassays) and was used to obtain a congenic strain with the LM-R *kdr* resistance allele introgressed into in a pyrethroid-susceptible background (section Isolation of the LMRKDR:RK strain).

### Isolation of the LKR strain

**Fig 1:**
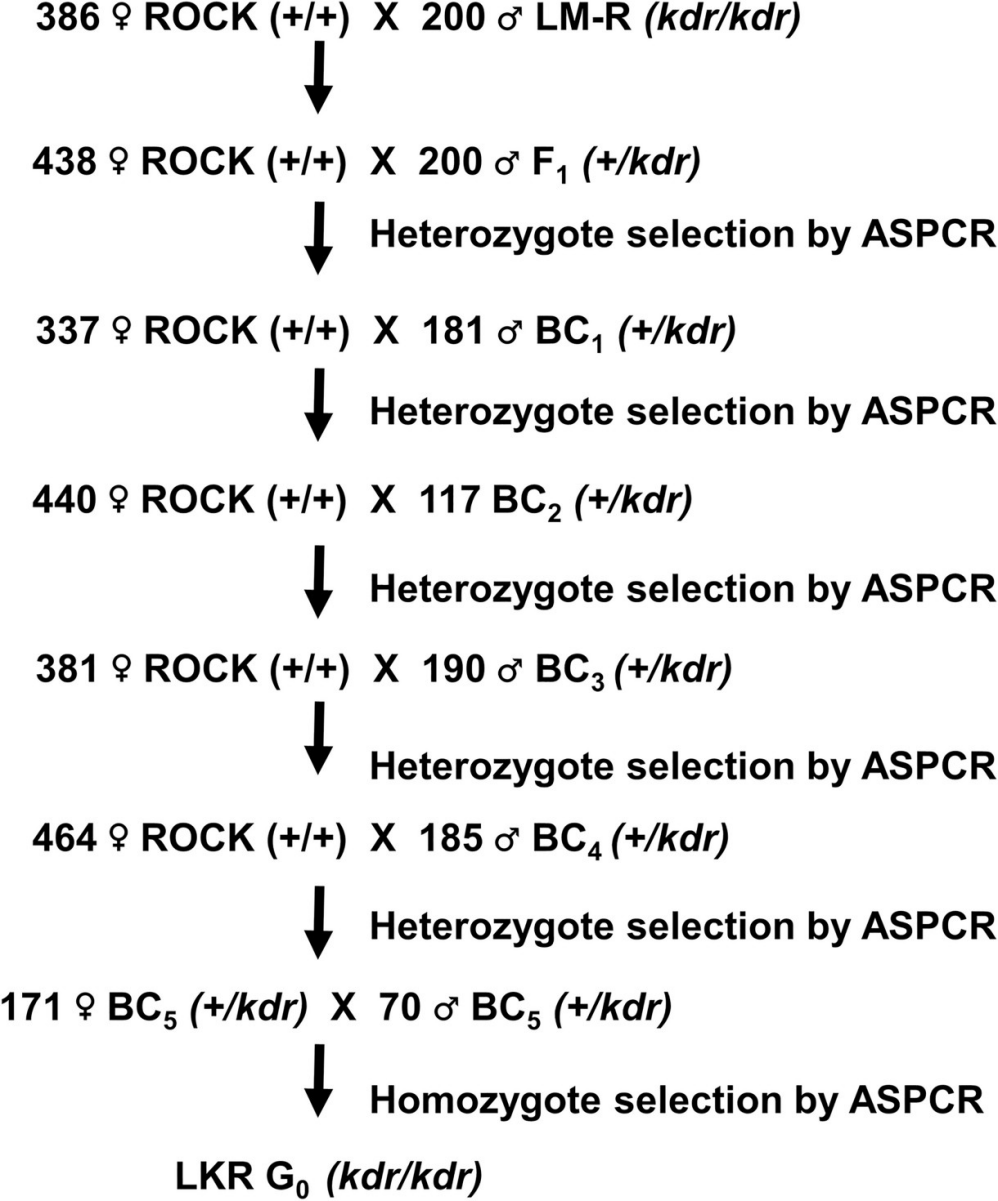
Protocol for the isolation of the congenic strain containing the 410L+1016I+1534C *kdr* allele introgressed into the genetic background of the ROCK strain. Number of heterozygous (+/*kdr*) males included in each backcross varied depending on the number of females added to the cage (approximately half the number of females).

To obtain the congenic LKR strain (having the *kdr* mutations from LM-R introgressed into the ROCK strain genome), unmated females of the susceptible ROCK strain were crossed with LM-R males and reared *en masse*. The F_1_ males were backcrossed with ROCK unmated females. The male progeny were genotyped for the V1016I *kdr* mutation (see next section) and heterozygotes were backcrossed with ROCK unmated females. This process was repeated another four times (Fig 1). Males and unmated females from the fifth backcross were genotyped. Heterozygotes were crossed *en masse*. The offspring from this cross were genotyped, and *kdr* homozygotes (30 males and 66 unmated females) were used to establish the new strain named LKR, which shares approximately 98.4% of its genomic background with ROCK. The genotype of individuals from the LKR strain were validated by Sanger sequencing of the three *kdr* mutations.

### Genotyping using allele specific polymerase chain reaction (ASPCR)

The genomic DNA from a hind tarsus of a mosquito leg was extracted using an alkali extraction method [25]. Legs were removed and placed into individual wells of a 96-well PCR plate (Bio-Rad, Hercules CA) with approximately three 2-3-mm diameter Zirconia/Silica beads (BioSpec Products, Bartlesville OK) and 10 μL of 0.2 M NaOH per well. Samples were vortexed for 1 minute to pulverize tissue and incubated at 70°C for 10 minutes. After incubation, 10 μL of neutralization buffer (360 mM Tris-HCl pH 8.0 and 10 mM EDTA) and 80 μL of nuclease-free water (VWR International, Radnor PA) were added to each well. Each mosquito was stored individually (no longer than 24 hours) in a P5000 tip (Gilson Inc., Middleton WI) stoppered with Kimwipe paper balls saturated with 10% sugar water until genotype was determined and heterozygotes were released in a cage.

To determine the genotype for V1016I, we did two allele specific PCR (ASPCR) reactions with gDNA extracted from each adult mosquito. One PCR reaction contained a primer specific for the susceptible allele (F1-V) and a reverse primer (M2-Rev) that is not allele specific. The second PCR reaction contained a primer specific for the resistance allele (F1-I) and the M2-Rev reverse primer. The PCR conditions used were: 94°C for 3 min, then 35 cycles of 94°C for 30s, 60°C for 30 s and 72°C for 1 min, followed by one cycle of 72°C for 7 min (36 cycles of amplification were used for PCR with the F1-V primer). PCR products were evaluated on 1% agarose gels. Adult males were considered heterozygous if ASPCR products were amplified with both sets of allele specific primers or homozygous if PCR products were amplified in only one of the two ASPCR reactions mentioned above. We validated the ASPCR primers for each PCR reaction by using control DNA from ROCK, LKR and heterozygotes (genotype determined by sequencing).

### RNA isolation, cDNA synthesis and sequencing of the full-length *Vssc* cDNA sequences from LKR and ROCK

Total RNA was extracted from 10 unmated 3-to-7-day old female mosquitoes of the LKR and ROCK strains using TRIzol Reagent (Invitrogen, Carlsbad CA) according to manufacturer’s instructions. Samples were treated with DNase I TURBO DNase (Life Technologies Co., Carlsbad CA). Total RNA concentration was determined using a NanoDrop 2000 (Thermo Fisher Scientific) and cDNA was synthesized using 5 μg of total RNA using the GoScript Reverse Transcription System (Promega, Madison WI) according to manufacturer’s instructions.

**Fig 2:**
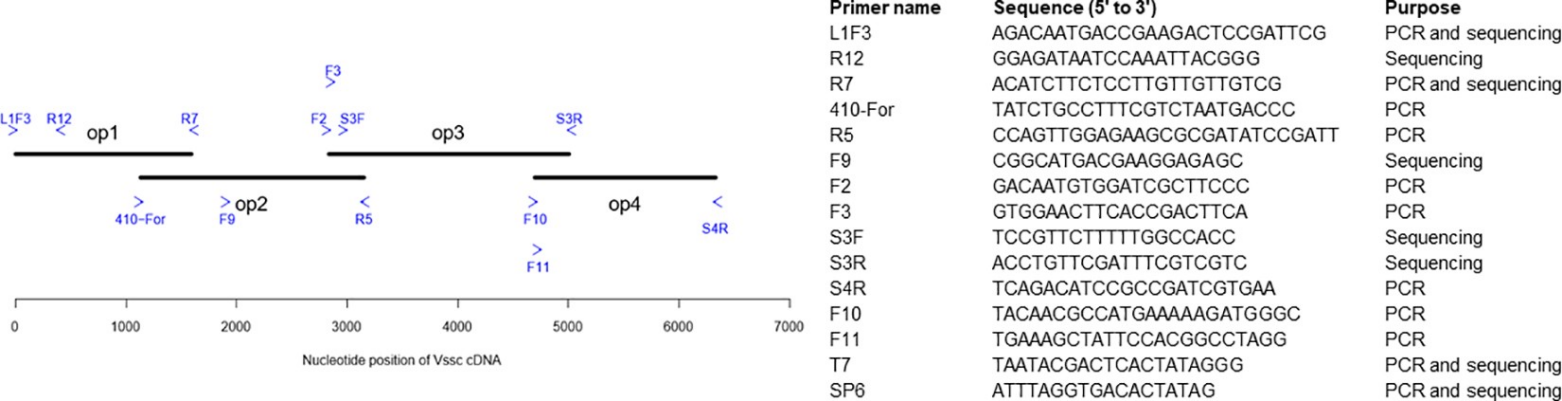
The *Vssc* cDNA from the ROCK and LKR strains was obtained by PCR and cloning of four overlapping fragments. The overlapping pieces and relative position of primers (blue arrows) are shown in the diagram. List of the primers, sequences and purposes are also included.

To obtain the full-length cDNA of *Vssc*, we amplified four overlapping pieces (also referred as op1-4) using PrimeStar GXL DNA high fidelity polymerase (Takara Bio Inc., Shiga, Japan) (Fig 2). For op1, L1F3 and R7 primers were used. For op2, 410-for and R5 primers were used. For op3, a PCR reaction was done using F2 and S3R primers and the products were used as templates for a nested PCR using F3 and S3R primers. For op4, a PCR reaction was done using F10 and S4R primers and the products were used as templates for a nested PCR using F11 and S4R primers. The overlap between op1-op2, op2-op3 and op3-op4 was approximately 393, 279 and 312 nucleotides, respectively. PCR reactions were done using Bio-Rad T100 Thermal Cycler (Bio-Rad, Hercules CA) under the following conditions: 95°C for 3 min, followed by 35 cycles of PCR (98°C for 10 s, 60°C for 15 s and 68°C for 4 min) and an extension at 68°C for 10 min. The PCR products from op2, op3 and op4 were gel excised from 1% agar gel stained with ethidium bromide under UV light. PCR products were purified using the Promega Wizard SV Gel and PCR Clean-up System (Promega, Madison WI). Purified cDNA fragments were inserted in pGEM-T Easy Vector (Promega, Madison WI) according to manufacturer’s instructions. Ligated plasmids were cloned into JM109 *Escherichia coli* competent cells (Promega, Madison WI). Transformed cells were grown on LB plus ampicillin in agar plates and incubated at 37°C overnight. Transformed colonies were assessed by PCR with primers T7 and SP6. PCR products were sequenced using primers R12, F9, S3R (Fig 2). Four clones of op2 and op3 from ROCK and LKR were sequenced. We had technical difficulties trying to clone op1, so instead the PCR products of op1 (from three independent reactions) were sequenced directly without cloning. Sequences were confirmed by multiple alignments using Clustal W in MegAlign Pro software (DNASTAR).

### Insecticides

**Fig 3:**
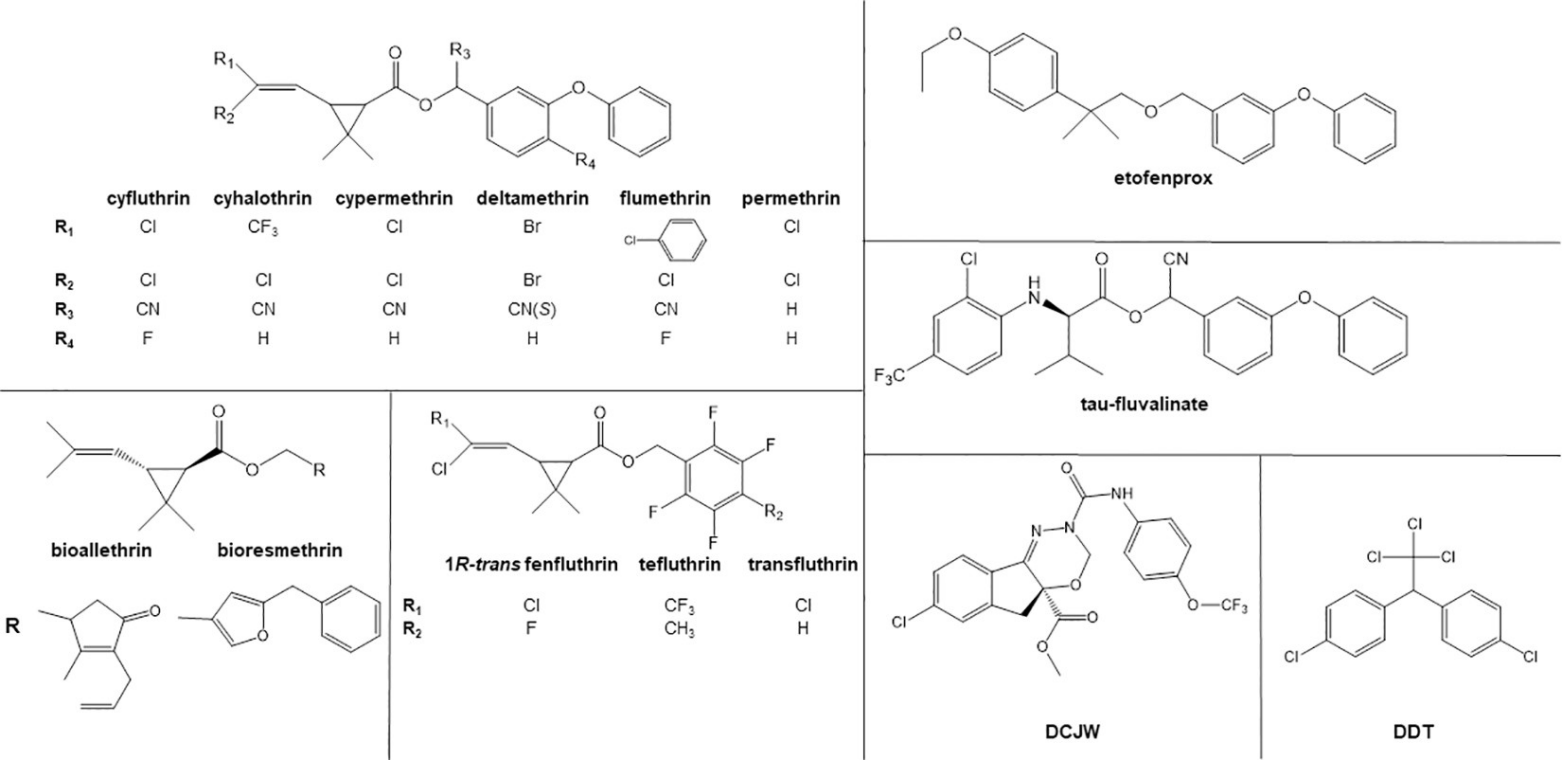
Structures of the insecticides tested. Stereochemistry is not indicated for all structures. All of the insecticides shown are pyrethroids, except for DDT and indoxacarb.

A total of 20 insecticides (representing diverse structures and known stereochemical composition) and three synergists were used (Fig 3): 1*R-cis αS* cypermethrin (100%, Bayer CropScience, Leverkusen, Germany), 1*R-trans* fenfluthrin (100%, Bayer CropScience), *alpha*-cypermethrin (99.5%, Chem Service, West Chester, PA, USA), bioallethrin (3.8% *cis* and 95.7% *trans*, Chem Service), bioresmethrin (99.5%, Chem Service), *cis*-permethrin (99.4%, Zeneca Ag products), cyfluthrin (99.2%, Chem Service), cyhalothrin (90.2%, ICI Americas), cypermethrin (44.8% *cis* and 53.9% *trans*, Chem Service), N-decarbomethoxylated JW062 (DCJW) (98%, DuPont), DDT (98%, Sigma-Aldrich, St. Louis, MO, USA), deltamethrin (99.5%, Chem Service), diethyl maleate (DEM) (97%, Sigma-Aldrich), etofenprox (96.3%, Mitsui Toatsu Chemicals, Tokyo Japan), flumethrin (100%, Bayer CropScience), permethrin (24% *cis* and 76% *trans*, Chem Service), piperonyl butoxide (PBO) (90%, Sigma-Aldrich), S,S,S-tributyl phosphorotrithioate (DEF) (97.3%, Chem Service), *tau*-fluvalinate (96%, Chem Service), tefluthrin (96%, Sigma-Aldrich), transfluthrin (99.5%, Chem Service), *trans*-permethrin (99%, FMC, Philadelphia, PA, USA).

### Adult female bioassays

We did insecticide bioassays for three purposes: to determine the levels of resistance to insecticides, to determine if enzyme-mediated detoxification is a major mechanism of resistance by using synergists, and to calculate the degree of dominance of pyrethroid resistance. To determine the levels of resistance to insecticides, solutions were dissolved in acetone and serial dilutions were prepared to find the range of doses that provided mortality values between 0% and 100%. Acetone was applied as control. Dosing of mosquitoes was conducted as outlined for cyhalothrin selections except, 20 adult females were treated per dose. Bioassays for each insecticide were repeated 5 times per strain. To determine if enzyme-mediated detoxification is a major mechanism of resistance in the LM-R strain, we conducted bioassays with cyhalothrin and the synergists PBO, DEM and DEF which inhibit CYPs, GSTs and hydrolases, respectively [30]. Bioassays were done as described above, except that 2.5 μg PBO, 5 μg DEM or 0.31 μg DEF (the maximal sublethal dose of each) were applied to the thorax of each adult female 1 hour prior to cyhalothrin application. For each replicate, controls included double acetone and an acetone plus synergist application.

We determined the degree of dominance of pyrethroid resistance in LKR relative to ROCK and KDR:ROCK to understand how pyrethroid resistance is inherited when the 410L + 1016I + 1534C allele is paired with a susceptible allele or the S989P + V1016G allele. To compare the dominance of resistance between LKR and ROCK, 400 unmated females of LKR were crossed with 200 ROCK males. Bioassays were done with adult females from the F_1_ using cyhalothrin (we chose cyhalothrin to calculate the degree of dominance because this is the pyrethroid used to select LM-R strains based on a previous study [31]. Reciprocal crosses were not conducted because *Vssc* is not sex linked [32]. The degree of dominance (D) was calculated according to Stone [33]. To compare the mode of inheritance between LKR and KDR:ROCK, 549 unmated females of KDR:ROCK strain were crossed with 220 males of LKR strain. Bioassays using permethrin and transfluthrin were done with F_1_ adult females and D was calculated as described above. We tested permethrin and transfluthrin because these two pyrethroids had some of the highest fold difference in the resistance ratios between KDR:ROCK and LKR (9.2- and 9.5-fold difference between KDR:ROCK and LKR, respectively).

For each insecticide, data from all replicates were pooled and assessed by probit analysis using R software version 3.6.3 [34] and a script that is publicly available (https://github.com/JuanSilva89/Probit-analysis/commit/2eaaff05da0f89294788bd0bed564e1bf257acf2) to determine the LD_50_ and 95% confidence intervals (CI 95%). Control mortality was corrected according to Abbott’s formula [35]. For each insecticide, the resistance ratios were calculated by dividing the LD_50_ of LKR by the LD_50_ of the ROCK strain. Statistical analysis between different resistance ratios was done as previously described [36].

## Results

### Selection of the LM strain using cyhalothrin

To reduce the presence of pyrethroid-susceptible alleles from LM-R strain, we conducted insecticide selections with cyhalothrin. An average of 2,790 virgin females and 1,164 males of LM strain were selected using cyhalothrin doses to kill approximately 80% of males and females (Table 1). We did not conduct more than three generations of selection because the LD_50_ of females between the second and third selections did not differ significantly which suggests that mosquitoes carrying cyhalothrin-susceptible alleles had been removed from the strain (CI 95% of LD_50_ for second and third selection were 6.8–8.5 and 6.4–8.8 ng cyhalothrin/female, respectively). Twelve individuals of LKR G_0_ were sequenced and all of them were homozygous for the three *kdr* mutations (V410L + V1016I + F1534C).

**Table 1:** Cyhalothrin selection of the LM strain.

Generation of selection	Sex	Dose (ng/mosquito)	Total individuals	Mortality (%)
1	Female	7.1	2941	85.2
	Male	1.8	1523	95.5
2	Female	10	2983	86.7
	Male	2.5	1048	78.7
3	Female	14	2445	85.2
	Male	5.0	920	85.4

### Synergist bioassays

The use of synergists DEF, DEM, or PBO in conjunction with cyhalothrin did not significantly decrease the levels of resistance in the LM-R strain (Table 2). These results suggest that enzyme-mediated detoxification by CYPs, GSTs or hydrolases are not the main mechanisms of resistance in LM-R strain.

**Table 2:** Bioassays using synergists and cyhalothrin to adult females of the ROCK and LM-R strains.

Synergist	Strain	LD_50_ ng/female (95% CI*)	RR_50_ (95% CI)
---	ROCK	0.049 (0.043–0.057)	
	LM-R	4.53 (4.27–4.81)	92.4 (74.9–112)
DEF	ROCK	0.014 (0.010–0.019)	
	LM-R	1.38 (1.19–1.61)	98.5 (62.6–161)
DEM	ROCK	0.024 (0.020–0.028)	
	LM-R	2.62 (2.44–2.81)	109 (87.1–141)
PBO	ROCK	0.005 (0.004–0.005)	
	LM-R	0.62 (0.52–0.74)	124 (103–186)

*95% CI: 95% Confidence Intervals.

### *Vssc* cDNA sequences

We found three non-synonymous mutations in the LKR strain that were different from the ROCK strain (V410L + V1016I + F1534C) based on the deduced amino acid sequence alignment using Clustal W. In addition, LKR has a fourth non-synonymous mutation (S723T), but this mutation is not associated with insecticide resistance as it is also found in the pyrethroid-susceptible NS strain [23]. We obtained two splice variants from the LKR strain (GenBank accession numbers MW202399 and MW202400 for LKR splice variants 1 and 2, respectively) and one splice variant from the ROCK strain (GenBank accession number MW202398). In LKR, splice variant 1 included exons b, e, f, h and i (but lacked exon a) whereas splice variant 2 included exons a, e and h (but lacked exon b, f and i). In ROCK, the splice variant included exons a, b and f (but lacked exons e, i and h).

### Levels of resistance in LKR

**Fig 4:**
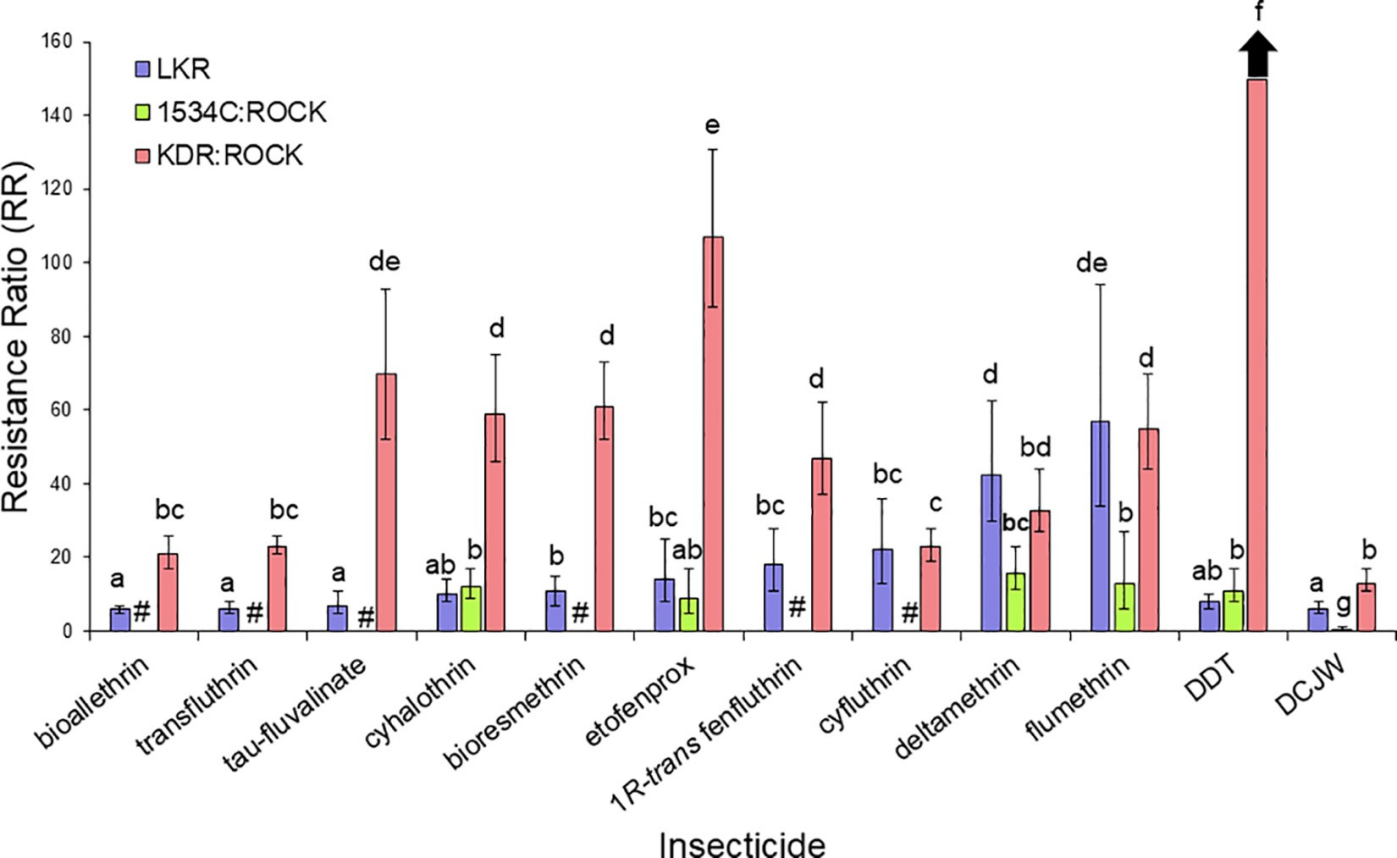
Levels of resistance levels conferred to insecticides targeting the VSSC in the LKR, 1534C:ROCK and KDR:ROCK strains having the 410L+1016I+1534C, 1534C and 989P+1016G alleles, respectively. RRs for KDR:ROCK are calculated from the values in Table 3 or were previously published [25] and are given here for comparison purposes. RRs for 1534C:ROCK for bioallethrin, cyhalothrin, etofenprox, flumethrin, DDT and DCJW were previously reported [24] and are given here for comparison purposes (those that were not tested are indicated by a #). Bars with different letters are significantly different based on non-overlap of the 95% confidence intervals.

**Fig 5:**
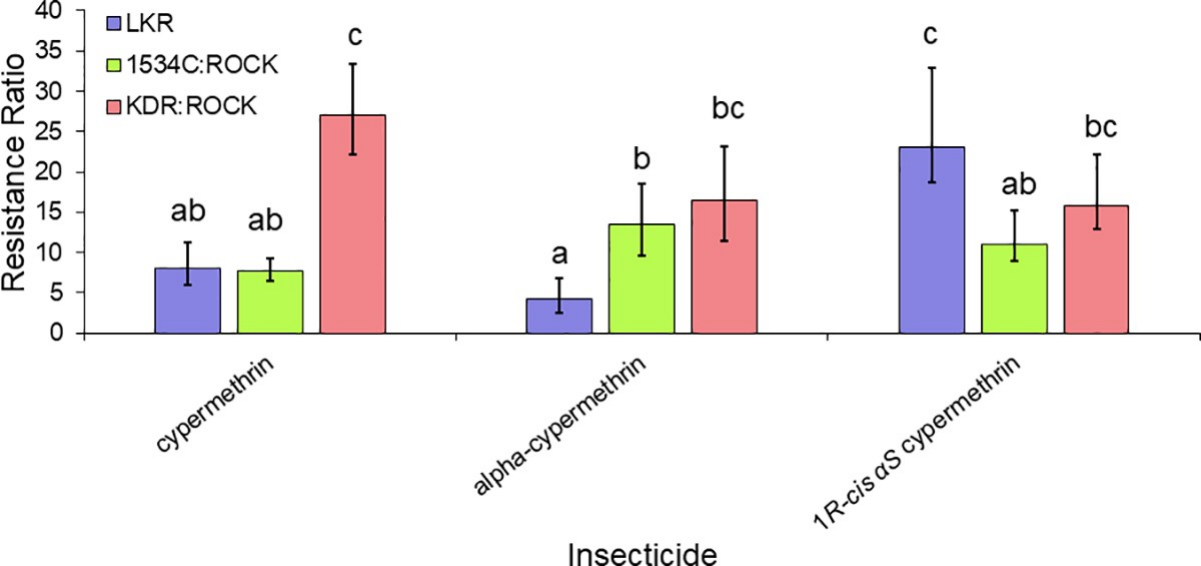
Levels of resistance levels conferred to 1*R-cis αS* cypermethrin, alpha-cypermethrin (mix of the 1*R-cis αS* and 1*S*-*cis αR* isomers), and cypermethrin (mix of eight isomers) in the LKR, 1534C:ROCK and KDR:ROCK strains having the 410L+1016I+1534C, 1534C and 989P+1016G alleles, respectively. The RR for KDR:ROCK to cypermethrin was previously published [25] and is given here for comparison purposes. Bars with different letters are significantly different based on non-overlap of the 95% confidence intervals.

**Fig 6:**
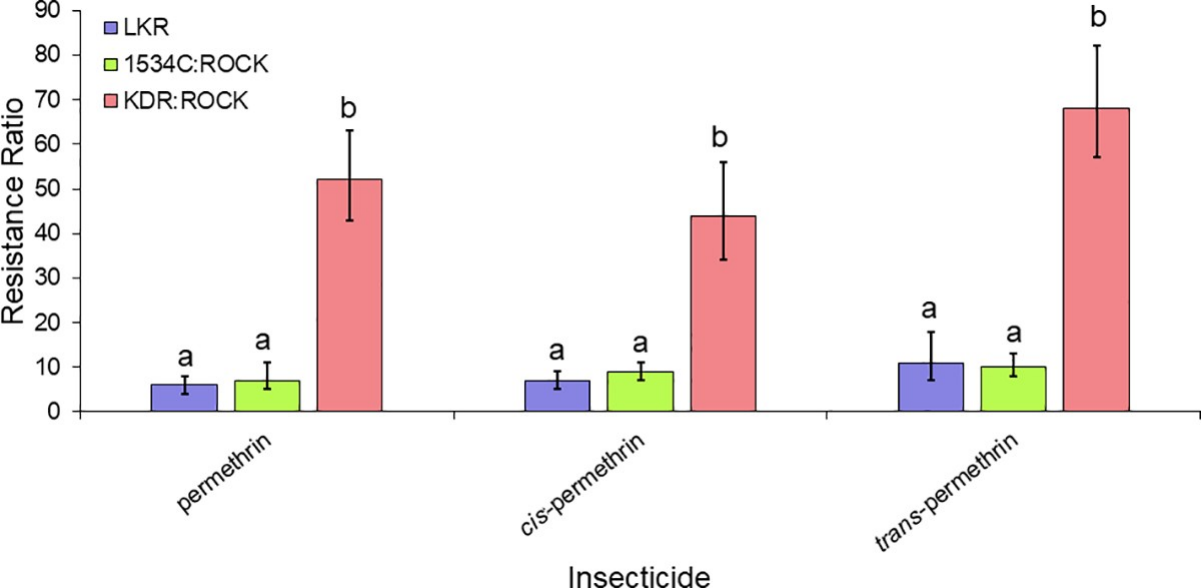
Levels of resistance levels conferred to permethrin, *cis-*permethrin, and *trans*-permethrin in the LKR, 1534C:ROCK and KDR:ROCK strains having the 410L+1016I+1534C, 1534C and 989P+1016G alleles, respectively. The RRs for KDR:ROCK to permethrin, *cis-*permethrin, and *trans*-permethrin were previously published [25] and given here for comparison purposes. Bars with different letters are significantly different based on non-overlap of the 95% confidence intervals.

**Fig 7:**
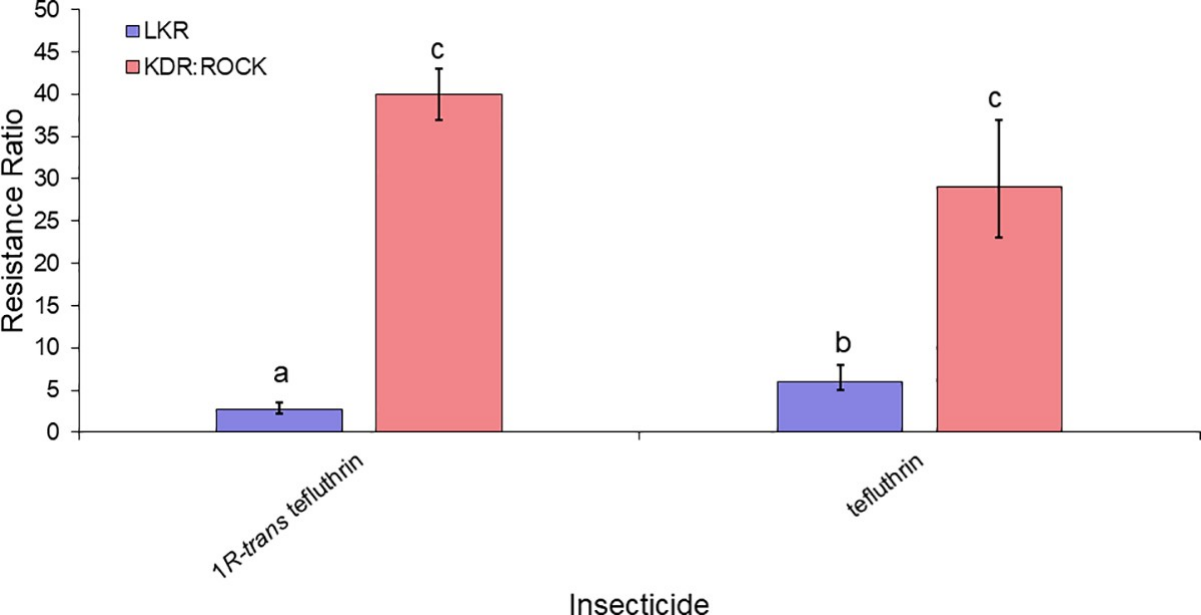
Levels of resistance levels conferred to tefluthrin and 1*R-trans* tefluthrin in the LKR and KDR:ROCK strains having the 410L+1016I+1534C and 989P+1016G alleles, respectively. The RR for KDR:ROCK to tefluthrin was previously published [25] and is given here for comparison purposes. Bars with different letters are significantly different based on non-overlap of the 95% confidence intervals.

The toxicity of 20 insecticides that target the sodium channel were determined against resistant and susceptible strains (Table 3). The levels of cross-resistance to pyrethroids, DCJW and DDT between LKR strain were compared in two general categories (the second category is subdivided in three groups of insecticides, see details below). The first comparison included insecticides with different structures, whereas the second group of comparisons was done to investigate the effect different stereoisomers had on resistance. For the first comparison, 15 insecticides (bioallethrin, bioresmethrin, cyfluthrin, cyhalothrin, cypermethrin, DCJW, deltamethrin, DDT, etofenprox, flumethrin, permethrin, tau-fluvalinate, tefluthrin, transfluthrin, and 1*R-trans* fenfluthrin) (Figs 4–7) were used. For these insecticides, resistance levels ranged from 6-fold (bioallethrin) to 57-fold (flumethrin) across the pyrethroids. There were no clear structural variations associated with higher or lower levels of resistance. LKR was also resistant to DCJW (6-fold) and DDT (8-fold). To evaluate the role of stereochemistry in the levels of resistance, we included three groups of insecticides having different isomers. The first group included 1*R-cis αS* cypermethrin, alpha-cypermethrin [mix of the (1*R*)-*cis* α*S* and (1*S*)-*cis* α*R* isomers], and cypermethrin (mix of eight isomers) (Fig 5). There was significantly higher resistance to 1*R*-*cis* α*S* cypermethrin than to cypermethrin or alpha-cypermethrin in LKR. The level of resistance in LKR was less than and greater than found in the 1534C:Rock strain for alpha-cypermethrin and 1*R-cis αS* cypermethrin, respectively. The results for the single enantiomer (1*R-cis αS* cypermethrin), were similar to the results for deltamethrin (Fig 1) which is also a single enantiomer and is structurally similar. The second set of insecticides included permethrin (mix of four isomers), *cis*-permethrin (mix of two isomers) and *trans-*permethrin (mix of two isomers). No significant differences were found between these insecticides in the LKR strain (Fig 6). In LKR, there was higher levels of resistance found for tefluthrin (mix of isomers, RR_50_ = 5), compared to 1*R-trans* tefluthrin (RR_50_ = 2.8).

**Table 3:** Toxicity of insecticides that target the VSSC against susceptible (ROCK) and resistant (1534C:ROCK, KDR:ROCK and LKR) strains of *A*. *aegypti*.

Insecticide^¥^	Strain	LD_50_* (95% CI)	Slope (±SEM)	*n*
1*R-cis-αS* cypermethrin	ROCK	0.008 (0.007–0.008)	3.0 (0.2)	680
	1534C:ROCK	0.088 (0.072–0.107)	2.3 (0.2)	506
	KDR:ROCK	0.12 (0.10–0.15)	2.1 (0.3)	520
	LKR	0.19 (0.15–0.23)	1.2 (0.1)	884
1*R*-*trans* fenfluthrin	ROCK	0.63 (0.53–0.75)	2.8 (0.3)	880
	LKR	11.1 (8.38–14.9)	1.8 (0.3)	600
1*R-trans* tefluthrin	ROCK	0.67 (0.65–0.70)	4.5 (0.1)	540
	KDR:ROCK	26.9 (25.8–28.2)	1.3 (0.1)	720
	LKR	1.87 (1.55–2.27)	1.3 (0.1)	680
alpha-cypermethrin	ROCK	0.032 (0.027–0.039)	3.5 (0.6)	817
	1534C:ROCK	0.43 (0.376–0.49)	2.5 (0.2)	578
	KDR:ROCK	0.53 (0.44–0.63)	1.8 (0.1)	712
alpha-cypermethrin	ROCK	0.048 (0.043–0.053)	2.7 (0.2)	520
	LKR	0.19 (0.13–0.29)	1.1 (0.1)	740
bioallethrin	ROCK	3.85 (3.61–4.11)	4.2 (0.2)	620
	LKR	21.9 (19.0–25.2)	2.7 (0.2)	420
bioresmethrin	ROCK	0.43 (0.392–0.472)	4.8 (0.4)	520
	LKR	4.54 (3.48–5.94)	1.0 (0.1)	820
*cis*-permethrin	ROCK	0.55 (0.54–0.57)	3.8 (0.1)	620
	1534C:ROCK	4.96 (4.07–6.05)	2.2 (0.3)	689
*cis*-permethrin	ROCK	0.70 (0.64–0.76)	1.5 (0.1)	480
	LKR	4.78 (4.06–5.64)	1.4 (0.1)	800
cyfluthrin	ROCK	0.023 (0.019–0.029)	2.1 (0.2)	640
	LKR	0.51 (0.38–0.69)	1.01 (0.1)	1100
cyhalothrin	ROCK	0.025 (0.023–0.026)	3.7 (0.2)	840
	LKR	0.25 (0.21–0.32)	1.4 (0.1)	760
	LKR x ROCK F1	0.050 (0.042–0.060)	2.2 (0.2)	620
cypermethrin	ROCK	0.075 (0.066–0.084)	3.2 (0.3)	837
	LKR	0.61 (0.50–0.74)	1.4 (0.1)	940
deltamethrin	ROCK	0.005 (0.004–0.006)	2.4 (0.4)	615
	LKR	0.21 (0.18–0.25)	1.1 (0.1)	820
etofenprox	ROCK	2.94 (2.85–3.04)	4.6 (0.1)	712
	LKR	42.2 (24.9–71.3)	1.2 (0.3)	700
flumethrin	ROCK	0.70 (0.59–0.83)	2.7 (0.3)	620
	LKR	39.7 (28.3–55.8)	0.7 (0.1)	1040
permethrin	ROCK	0.23 (0.23–0.24)	3.6 (0.04)	800
	LKR	1.30 (0.94–1.81)	1.15 (0.09)	814
	KDR:ROCK	12.1 (10.2–14.3)	4.4 (0.3)	660
	KDR:ROCK x LKR F1	10.5 (9.01–12.3)	2.2 (0.2)	520
tau-fluvalinate	ROCK	0.69 (0.59–0.82)	3.2 (0.5)	440
	LKR	4.99 (3.94–6.34)	1.1 (0.1)	880
tefluthrin	ROCK	4.97 (4.62–5.33)	4.2 (0.3)	540
	LKR	25.2 (19.5–32.5)	2.1 (0.4)	720
transfluthrin	ROCK	0.63 (0.60–0.66)	4.8 (0.2)	520
	LKR	3.83 (3.17–4.63)	1.6 (0.1)	540
	KDR:ROCK	14.8 (13.8–15.8)	3.1 (0.2)	520
	KDR:ROCK x LKR F1	11.3 (9.37–13.6)	2.0 (0.2)	571
*trans*-permethrin	ROCK	0.45 (0.41–0.49)	3.8 (0.3)	513
	1534C:ROCK	4.45 (3.79–5.21)	3.2 (0.2)	520
*trans*-permethrin	ROCK	0.57 (0.48–0.67)	3.7 (0.5)	900
	LKR	6.22 (4.37–8.86)	1.0 (0.1)	520
DCJW	ROCK	1.84 (1.72–1.96)	4.0 (0.2)	720
	LKR	11.3 (9.63–13.3)	1.9 (0.2)	727
DDT	ROCK	15.7 (15.2–16.3)	3.8 (0.1)	640
	LKR	118 (96.7–144)	1.4 (0.1)	994

^¥^Bioassays done with the same insecticide solutions for different strains share the same insecticide name.

*LD_50_, ng/mosquito.

We also determined the inheritance of pyrethroid resistance in LKR relative to a susceptible strain and a resistant strain (ROCK and KDR:ROCK, respectively). Relative to ROCK strain, the inheritance of cyhalothrin resistance in LKR is incompletely recessive (D = -0.42). In crosses with the KDR:ROCK strain, resistance was inherited as incompletely recessive traits, with D values of -0.87 (permethrin) and -0.60 (transfluthrin).

## Discussion

Our isolation of a congenic resistant strain having the 410L+1016I+1534C *kdr* allele allowed us the opportunity to compare resistance levels of two other *kdr* alleles (1534C and 989P+1016G) that had been isolated in the same genetic background [24,25]. Relative to the 1534C allele, the 410L+1016I+1534C allele conferred similar levels of resistance relative to seven insecticides (cyhalothrin, cypermethrin, etofenprox, DDT, cypermethrin, permethrin, *cis-*permethrin and *trans-*permethrin), conferred higher levels of resistance to flumethrin, DCJW, 1*R*-*cis* α*S* cypermethrin and deltamethrin), and conferred lower levels of resistance only to alpha-cypermethrin (Figs 4–5). The similar resistance levels conferred to cyhalothrin, cypermethrin, etofenprox, DDT, cypermethrin, permethrin, *cis-*permethrin and *trans-*permethrin by the 410L+1016I+1534C and 1534C alleles suggests that resistance to these insecticides is conferred by the 1534C mutation, with the V410L and/or the V1016G mutations adding nothing to the resistance. In contrast, the 410L+1016I+1534C allele did confer higher levels of resistance to flumethrin, DCJW, 1*R-cis αS* cypermethrin and deltamethrin, relative to the F1534C allele, suggesting addition of the V410L and/or V1016I mutations to F1534C enhances resistance to these insecticides. Comparisons of the 989P+1016G (KDR:ROCK) and 410L+1016I+1534C (LKR) alleles revealed that there were higher levels of resistance conferred by the 989P+1016G allele for eleven of the insecticides tested. For cyfluthrin, flumethrin, alpha-cypermethrin, 1*R*-*cis* α*S* cypermethrin and deltamethrin the resistance levels were not significantly different between these alleles. The importance of pyrethroid stereochemistry to the resistance levels is exemplified by the higher level of resistance to 1*R*-*cis* α*S* cypermethrin in LKR, relative to cypermethrin (mix of 8 isomers) or alpha-cypermethrin, Fig 5). This pattern was not observed for either the 1534C or the 989P+1016G alleles.

There are non-synonymous mutations in *Vssc* that do not cause resistance [37] making it necessary to evaluate the resistance of each allele found. In some cases, sequential accumulation of mutations can give rise to alleles that give greater levels of resistance. The best example of this is house flies, where the first *Vssc* mutation was L1014F (*kdr*). Subsequently a second mutation (M918T) evolved, giving rise to a new allele (918T+1014F or *super-kdr*) which gave higher levels of resistance to most pyrethroids [38]. A subsequent mutation gave rise to a new allele 600N+918T+1014F giving even higher levels of resistance [39,40]. In contrast, the levels of resistance conferred to pyrethroids by the 410L+1016I+1534C allele is quite modest (2.8- to 57-fold) and not greater than the 1534C allele for six of the 10 pyrethroids for which comparisons were possible. Thus, it is unclear why these two alleles are among the most common [41] and what evolutionary forces selected for the triple mutation allele. The only widely used pyrethroid for which the 410L+1016I+1534C allele gives more resistance is deltamethrin (Figs 4–7). Unfortunately, we do not know enough about use patterns of pyrethroids to know if deltamethrin use is the selective advantage favoring the 410L+1016I+1534C allele (over the 1534C allele) or not. A second possibility is that the 410L+1016I allele was present in a population, the F1534C mutation occurred in the 410L+1016I background to generate the triple mutation allele, and the selective advantage of the allele to most pyrethroids is simply due to the 1534C mutation. A third possibility is that the V410L and/or V1016I mutations do not contribute to insecticide resistance, but ameliorates the fitness cost of the 1534C mutation. Previous studies in Latin America found that the 410L+1016I+1534C allele has higher frequencies relative to the 410L+1534C allele. [16,42].

How *kdr* mutations alter VSSC sensitivity to pyrethroids has been tested by heterologous expression in *Xenopus* oocytes. However discrepancies between oocytes and *in vivo* results have been previously observed [24,38] and our results add additional discrepancies. For example, In heterologous expression studies, the F1534C mutation conferred insensitivity to permethrin, but not deltamethrin [26,43]. However, the 1534C allele confers resistance to both permethrin and deltamethrin *in vivo* [24,44]. Herein, we show the 410L+1016I+1534C allele confers similar levels of resistance to the 1534C allele even though heterologous expression studies found that the 410L+1534C mutations conferred higher insensitivity to permethrin than F1534C mutation alone [15]. Although we measured the resistance levels in the 410L+1016I+1534C allele, our results show that the addition of V410L to F1534C did not increase the levels of resistance to permethrin (or *cis*-permethrin or *trans*-permethrin). Overall, these discrepancies support the need to quantify resistance conferred by different *kdr* mutations *in vivo*. The possibilities here are now quite large, as use of genome editing tools such as CRISPR/Cas9 to *knock in* the *kdr* mutations allows opportunities to explore mutations and combinations of mutations that are not necessarily found in nature. Isolation of congenic strains with different *kdr* mutations also allows for studies on the impacts of these mutations on multiple aspects of mosquito biology (e.g. fitness costs).

Different *kdr* alleles are more likely to increase in frequency within populations under pyrethroid selection depending on the resistance levels they confer. For example, the 989P+1016G allele is likely to increase in frequency within populations that have the 410L+1016I+1534C or 1534C alleles in environments exposed to permethrin and transfluthrin, and this has been observed [45]. Knowing the levels or resistance conferred by different *kdr* alleles allows vector control personnel to choose the pyrethroid having the lowest levels of resistance for the alleles found in their region.

In this study we identified the main mechanism of pyrethroid-resistance in a strain of *A*. *aegypti* collected in Colombia to be *kdr* (410L+1016I+1534C allele), and quantified the levels of resistance to different insecticides targeting VSSC that this allele confers. We found that resistance conferred by this *kdr* allele can vary 10-fold between pyrethroids, and can vary between stereoisomers of a pyrethroid as well. We propose that maximum resolution of experiments aimed at understanding how *kdr* mutations alter VSSC sensitivity will be achieved using with individual pyrethroid diastereomers. We also found that levels of resistance conferred *in vivo* do not match the results of studies where the validation of *kdr* mutations was done through heterologous expression. We suggest that genome editing techniques such as CRISPR-Cas9, to generate single or multiple mutations, will help to provide high quality determinations of the effects mutations have on VSSC sensitivity to insecticides. We found that the 410L+1016I+1534C *kdr* allele does not confer higher levels of resistance to six out of 10 pyrethroids relative to the resistance levels conferred by the F1534C allele. These results improve our understanding of the evolution of insecticide resistance and are useful for insecticide resistance management because we show which insecticides are most and least effective at controlling mosquitoes carrying the 410L+1016I+1534C.
